# Efficacy of epidural steroid injection in the treatment of sciatica secondary to lumbar disc herniation: a systematic review and meta-analysis

**DOI:** 10.3389/fneur.2024.1406504

**Published:** 2024-05-22

**Authors:** Jianan Zhang, Ruimeng Zhang, Yue Wang, Xiaoqian Dang

**Affiliations:** ^1^Zonglian College, Xi’an Jiaotong University, Xi’an, China; ^2^The Second Affiliated Hospital of Xi’an Jiaotong University, Xi’an, China

**Keywords:** epidural injection, steroid, sciatica, lumbar disc herniation, meta-analysis

## Abstract

Epidural steroid injection for the treatment of sciatica caused by disc herniation is increasingly used worldwide, but its effectiveness remains controversial. The review aiming to analyze the efficacy of epidural steroid injection on sciatica caused by lumbar disc herniation. Randomized controlled trials (RCTs) investigating the use of epidural steroid injections in the management of sciatica induced by lumbar disc herniation were collected from PubMed and other databases from January, 2008 to December, 2023, with epidural steroid injection in the test group and epidural local anesthetic and/or placebo in the control group. Pain relief rate, assessed by numerical rating scale (NRS) and visual analogue scale (VAS) scores, and function recovery, evaluated by Roland Morris Disability Questionnaire (RMDQ) and Oswestry Disability Index (ODI) scores, were recorded and compared. Meta-analysis was performed by Review Manager. In comparison to the control group, epidural steroid injections have been shown to be effective for providing short- (within 3 months) [MD = 0.44, 95%CI (0.20, 0.68), *p* = 0.0003] and medium-term (within 6 months) [MD = 0.66, 95%CI (0.09,1.22), *p* = 0.02] pain relief for sciatica caused by lumbar disc herniation, while its long-term pain-relief effect were limited. However, the administration of epidural steroid injections did not lead to a significant improvement on sciatic nerve function in short- [MD = 0.79, 95%CI = (0.39, 1.98), *p* = 0.19] and long-term [MD = 0.47, 95% CI = (−0.86, 1.80), *p* = 0.49] assessed by IOD. Furthermore, the analysis revealed that administering epidural steroid injections resulted in a reduction in opioid usage among patients with lumbar disc herniation [MD = −14.45, 95% CI = (−24.61, −4.29), *p* = 0.005]. The incidence of epidural steroid injection was low. Epidural steroid injection has demonstrated notable efficacy in relieving sciatica caused by lumbar disc herniation in short to medium-term. Therefore, it is recommended as a viable treatment option for individuals suffering from sciatica.

## Introduction

Sciatica is characterized by pain along the path and distribution area of the sciatic nerve (1). It typically presents as radiating pain in the buttocks, back of the thigh, posterolateral calf, and lateral dorsum of the foot. Most cases of sciatica are caused by problems with the structures surrounding the sciatic nerve, resulting in irritation, compression, and damage to the nerve, which is referred to as secondary sciatica (1, 2). The main reason for this is typically lesions caused by lumbar disc herniation, while a smaller percentage is due to primary sciatic neuritis (3, 4). Lumbar intervertebral disc herniation is the primary cause of sciatica. This condition typically results from the progressive deterioration of the lumbar intervertebral disc, exacerbated by daily external pressure and strain. This leads to the rupture of the annulus fibrosus, allowing the nucleus pulposus to protrude and exert pressure on the adjacent lumbar nerve roots or the cauda equina nerve. This phenomenon commonly occurs at the lumbar 4–5 and lumbar 5-sacral 1 segments (5). Currently, the main approach to managing sciatica caused by lumbar disc herniation involves the use of anti-inflammatory, anti-edema, analgesic, and neurotrophic medications (5–7). During the acute phase, mannitol might be utilized for dehydration to reduce swelling and alleviate nerve pressure, thus relieving symptoms. Furthermore, the combination of mannitol with anti-inflammatory medications, particularly non-steroidal drugs such as aspirin and celecoxib, could assist in pain relief by suppressing the inflammatory response. Moreover, neurotrophic medications have shown promise in enhancing pain relief. Presently, the effectiveness of drug treatments is deemed unsatisfactory, although patients with mild illnesses might experience temporary clinical improvements, those with severe conditions or recurring episodes often only achieve short-term symptom relief with medication (7). Regrettably, once the drug is ceased, symptoms might relapse, impeding the attainment of long-term pain relief (6, 8).

Steroid hormones have proven to be effective in preventing and reducing inflammation, edema, and nerve root compression (9). As a result, they offer a viable alternative for alleviating sciatica that occurs due to the inflammatory response caused by lumbar disc herniation (10, 11). Epidural steroid injection is utilized as a treatment option for back pain and radiation of radicular pain, they function by reducing inflammation that impacts the epidural nerve tissue, thereby decreasing nerve fiber damage and partially thwarting lumbar disc herniation (11–13). Furthermore, they aid in blocking pain signals transmitted by medium C nerve fibers. A previous conducted study revealed that transforaminal epidural steroid injection could provide significant relief for sciatica, with the effects lasting up to 12 months, which were consistent with positive outcomes of surgical interventions for sciatica, as a result, this study posited epidural steroid injections as a viable treatment option for sciatica, suggesting that alternative treatments might yield even more favorable results (14). According to another study, epidural steroid injection was shown to provide more effective relief for sciatica when compared to traction, exercise therapy, and drug therapy (15). Therefore, epidural steroid injection is increasingly being used to treat sciatica resulting from lumbar disc herniation. There are three approaches for epidural corticosteroid administration: interlaminar, transforaminal, and caudal. Recent reviews reveal that transforaminal and parasagittal interlaminar ESI providing better outcomes than interlaminar injections (16–18).

There is currently a continuing debate within the medical community regarding the effectiveness of epidural steroid injection for the treatment of sciatica induced by lumbar disc herniation. According to a meta-analysis conducted in 1995, which reviewed 13 RCTs, it was found that epidural steroid injection could be a beneficial option for improving symptoms of patients with sciatica (19). However, A systematic review conducted by Rafael Zambelli Pinto in 2012, which analyzed 25 published reports and 23 clinical trials, found that these injections did not have a notable effect on alleviating pain or decreasing long-term disability in comparison to placebo treatments (20). Although these injections might provide momentary pain relief, it is crucial to note that they do not lead to considerable enhancement in lower extremity function as time progresses. According to a 2015 study, after examining numerous sciatica patients, researchers found that epidural corticosteroid injections did not provide significant relief in both the short and long term, additionally, it was noted that epidural steroid injection could potentially lead to adverse effects (21). In 2019, a meta-analysis of 25 clinical trials found moderate-quality evidence that epidural corticosteroid injections may be slightly more effective in reducing leg pain and disability at short-term follow-up, and the treatment effect was small and may not be considered clinically important (22). Nonetheless, A meta-analysis of 6 RCTs has shown that the use of ESI is more effective for alleviating lumbosacral radicular pain than conservative treatments in terms of short-term and intermediate-term (23). In the same year, a comprehensive systematic review come to the conclusion that there is strong evidence that lumbar transforaminal injection of steroids is an effective treatment for radicular pain due to disc herniation (24). A meta-analysis conducted in 2021 incorporated 17 RCTs that indicated a significant reduction in leg pain and improvement in function at the six-week mark for individuals with sciatica who received epidural steroid injection compared to those given a placebo injection (25). Another meta-analysis conducted in August of the same year, which analyzed 21 RCTs with a follow-up of at least 6 months, clearly demonstrated the effectiveness of combining local anesthesia with steroids (26). In 2022, a meta-analysis of 25 RCTs found that steroid treatment was superior to local anesthesia or placebo in improving outcomes at 1 and 3 months, however, there was no significant difference between using local anesthesia alone versus local anesthesia combined with steroids in enhancing the function of the affected limb (27). In 2024, an Evidence-based review show that multiple randomized controlled trials and high-quality observational studies provide varying degrees of evidence supporting the efficacy of ESI compared to placebo in reducing pain and improving function (18).

These results suggested that the effectiveness of epidural steroid injection for alleviating neuralgia resulting from lumbar disc herniation is still uncertain. Further research is needed to elucidate the clinical efficacy of this treatment. As such, the current study included recent RCTs that compared epidural corticosteroid injection to placebo and/or local anesthetics for treating sciatica. Moreover, the up-to-date RCT (28) found that the epidural steroid injection significantly reduces the use of opioids than solely usual care at long term, but they did not pay attention to the doses. So our study examined the effect of epidural injections on the dose of opioids administered. This meta-analysis utilized more stringent inclusion criteria to provide a clearer evaluation of the effectiveness.

## Materials and methods

### Literature search strategy

We obtained literature on the efficacy of epidural steroid injections for treating sciatica caused by lumbar disc herniation by searching several databases, including Pubmed, Embase, Web of Science, and the Cochrane Library. The search was conducted from January 1, 2008 to December 31, 2023. We used a combination of specific subject terms and general keywords such as sciatica, epidural steroids, lumbar disc herniation, and randomized controlled trial. Additionally, we reviewed the references of the included studies to ensure a comprehensive search outcome. The search strategy was available in Supplementary File.

### Main outcomes

The main outcome was pain relief, which was assessed mainly by numerical rating scale (NRS) and visual analogue scale (VAS) scores. Secondary outcomes were: (1) function recovery, mainly assessed by Roland Morris Disability Questionnaire (RMDQ) and Oswestry Disability Index (ODI) scores; (2) opioid dose; (3) adverse effects related to drugs used for sciatica treatment.

NRS scale: 0 (no pain), 1–3 (mild pain), 4–6 (moderate pain) and 7–10 (severe pain);

VAS scale: 0 (no pain), 3 or less (mild pain), 4–6 (pain and interfering with sleep, tolerable), 7–10 (intense pain, intolerable, interfering with appetite and sleep);

ODI scale: consists of 10 questions, the higher the score, the more severe the dysfunction;

RMDQ scale: minimum 0 points, maximum 24 points, the higher the score, the higher the degree of dysfunction.

### Inclusion and exclusion criteria

Inclusion criteria: (1) the study was an RCT; (2) patients with sciatica, which was confirmed by examination to be caused by lumbar disc herniation, with no restriction on age or gender; (3) the intervention involved administering steroid injections through one of three approaches: caudolateral, interlaminar, or intervertebral foraminal. The control group received epidural injections of a local anesthetic and/or a placebo; (4) The eligible studies included in the analysis provided accurate and comprehensive statistical data, which covered at least one outcome measure such as pain relief, assessment of function, or opioid use.

Exclusion criteria: (1) literature not in English; (2) literature for which full text is not available; (3) repeatedly published literature; (4) review literature, conference abstracts, case reports, etc.; (5) sciatica not caused by lumbar disc herniation; (6) patients with a history of previous lumbar disc surgery and postoperative epidural steroid injections; (7) associated neuralgia caused by cervical or thoracic disc herniation; (8) animal experiments.

### Data selection and extraction

Three authors imported the selected literature into Endnote X9 software, looked for duplicates and removed duplicate items. Two authors performed literature screening independently. They firstly read the titles and abstracts of the literature to exclude a portion of the literature and then read the full text of the remaining literature to determine the literature to be included in the meta-analysis based on the inclusion and exclusion criteria. If there was a disagreement, they consulted the third author to decide whether the literature was included in the analysis or not. The extraction information of literatures included first author, year of publication, number of subjects, intervention, dosage of steroids and local anesthetics, NRS score and/or VAS score, and clinical outcomes.

### Quality assessment

The Cochrane Quality Assessment Criteria in Revman 5.4 was used to evaluate the risk of bias in this study. Two authors independently assessed the literature included in the study and compared their results. In case of any disagreement, the third author was consulted for resolution. The evaluation focused on seven important factors: randomized sequence generation, allocation concealment, blinding of participants and staff, blinding of outcome assessors, incomplete outcome data, selective reporting, and bias from other sources. Each of these factors was categorized into three outcomes: “high risk,” “low risk,” and “unknown risk.”

### Statistical analysis

Statistical analysis was performed on Review Manager 5.4 software. The quantitative data were statistically analyzed by calculating weighted mean differences (MD) and 95% confidence intervals (CI). Multiple included studies were tested for heterogeneity, and *I^2^* values and *p-*values were calculated to determine whether they were homogeneous and to select a fixed-effects model or a random-effects model. When *I^2^* < 50%, the heterogeneity among multiple studies was considered not statistically significant, and the fixed-effects model was used to calculate the combined statistics, and when *I^2^* ≥ 50%, the heterogeneity among multiple studies was considered statistically significant, and the random-effects model was used to calculate the combined statistics, and sensitivity analyses were performed to find the source of heterogeneity. Funnel plot was used to detected the presence or absence of publication bias. *p*-value <0.05 was consider to be statistically significant.

## Result

### Study selection

**Figure 1:**
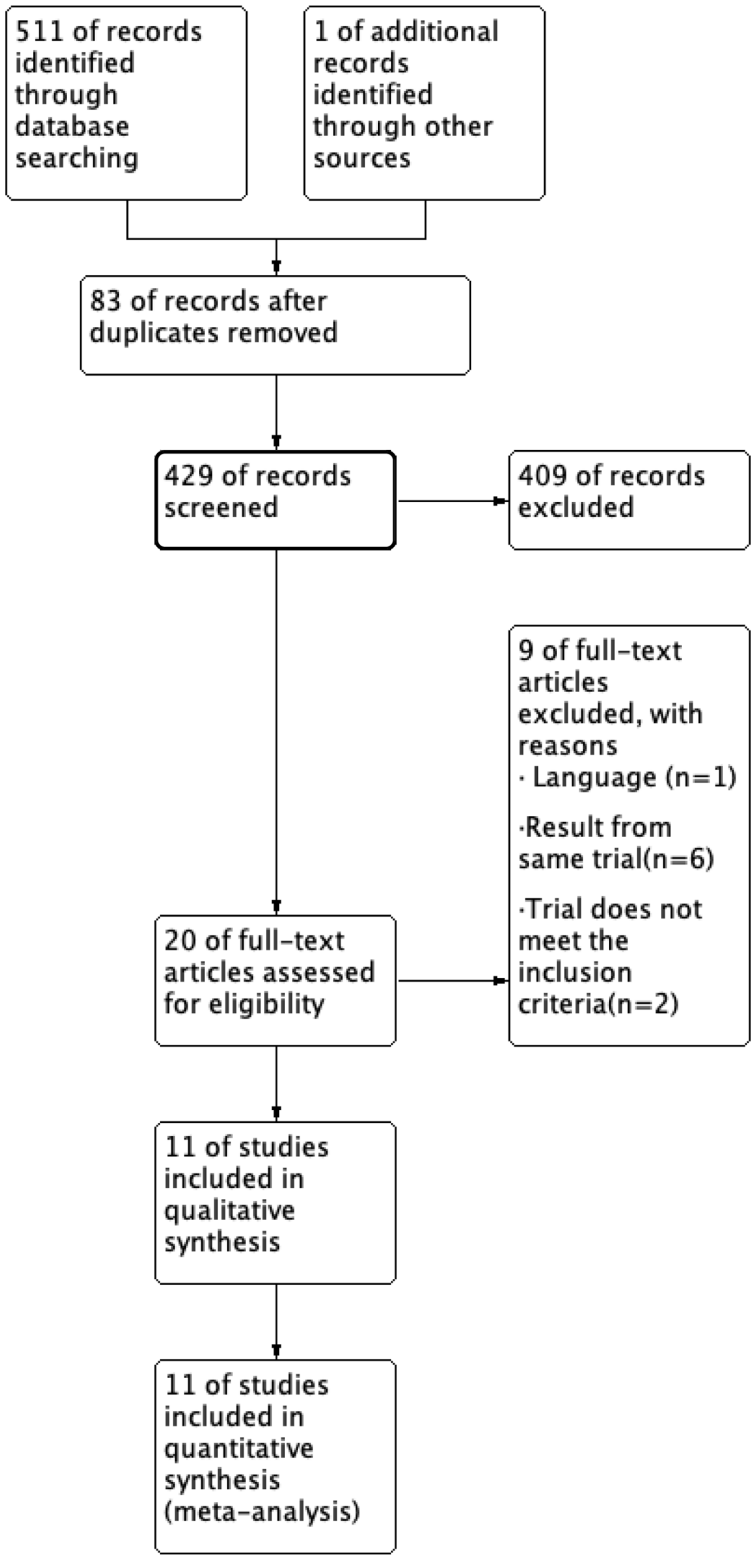
Flow diagram of study screening and selection.

The initial phase of the study involved gathering 511 retrieval records along with 1 additional record. Subsequently, 83 duplicate articles were removed, resulting in a total of 429 articles for analysis. Upon careful assessment of the titles and abstracts, 409 of the articles were deemed unsuitable and excluded from further consideration. Finally, after a thorough examination of the full text, 11 articles were selected for inclusion in the study (Figure 1), adhering to the specific criteria established for inclusion and exclusion. The details of included studies were summarized in Table 1. Among the 11 studies analyzed, the earliest RCT was published in 2008, 7 RCTs were published from 2012 to 2017, with 2 studies specifically in 2012, this suggested that the topic has gained increasing attention starting from 2012. And 2 studies were published in recent 2 years.

**Table 1:** Details of included study.

**Study**	**Year**	**Number**	**Duration**	**Intervention**	**Control**	**Outcome**	**Administration route**	**Proportion of people with pain reduction of more than 50% in intervention group**	**Proportion of people with pain reduction of more than 50% in control group**
Chae et al.	2022	Intervention: 24 Control: 26	4 weeks	8 ml injections of 0.33% lidocaine with 5 mg of dexamethasone	8 ml injections of 0.33% lidocaine	VAS, RMDQ, Adverse event	Transforaminal	–	–
Ghai et al.	2015	Intervention: 35 Control: 34	2 weeks, 1, 2, 3, 6, 9, and 12 months	8 ml of 0.5% lidocaine	6 ml of 0.5% lidocaine mixed with 80 mg (2 ml) of methylprednisolone acetate	NRS, MODQ, Adverse event	Parasaggital interlaminar	86%	50%
Manchikanti et al.	2008	Intervention: 42 Control: 42	3, 6, and 12 months	9 ml of lidocaine with 1 ml of steroid, followed by 2 ml of 0.9% sodium chloride solution as a flush.	10 ml of lidocaine 0.5%, followed by 2 ml of 0.9% sodium chloride solution as a flush.	NRS, ODI, adverse event, Opioid use	Caudal	81%	81%
Manchikanti et al.	2010	Intervention: 35 Control: 35	3, 6, and 12 months	0.5% lidocaine, 5 ml, mixed with 1 ml of non-particulate betamethasone.	Lidocaine 0.5%, 6 ml	NRS, ODI, adverse event, Opioid use	Interlaminar	86%	74%
Manchikanti et al.	2012	Intervention: 60 Control: 60	3, 6, and 12 months	9 ml of lidocaine mixed with 6 mg of betamethasone (either brand name or non-particulate) or 40 mg of methylprednisolone	10 ml of lidocaine hydrochloride 0.5%	NRS, ODI, adverse event, Opioid use	Caudal	80%	77%
Manchikanti et al.	2013	Intervention: 60 Control: 60	3, 6, and 12 months	6 ml derived from preservative-free lidocaine 0.5%, 5 ml, mixed with 1 ml of 6 mg non-particulate betamethasone	0.5% preservative-free lidocaine 6 ml	NRS, ODI, adverse event, Opioid use	Interlaminar	72%	67%
Manchikanti et al.	2014	Intervention: 60 Control: 60	3, 6, and 12 months	preservative-free lidocaine 1% followed by 3 mg of betamethasone, either particulate or non-particulate.	1.5 ml of preservative-free lidocaine 1%, followed by a 0.5 ml sodium chloride solution	NRS, ODI, adverse event, Opioid use	Transforaminal	73%	77%
Nandi et al.	2017	intervention:47 control:46	4 and 12 weeks	20 ml steroid solution (methyl prednisolone 80 mg diluted in 18 ml of isotonic saline)	20 ml of isotonic saline	VAS, ODI, RMDQ	Caudal	60%	48%
Ökmen et al.	2016	Intervention: 48 Control: 50	1, 3, 6, 12 months	5 ml bupivacaine +1 ml 40 mg methylprednisolone acetate +4 ml sterile saline.	5 ml bupivacaine +5 ml sterile saline	VAS, ODI	Transforaminal	–	–
Steve et al.	2012	Intervention: 28 Control: 30	4 weeks	60 mg of methylprednisolone acetate plus 0.5 ml of saline for a total volume of 2 ml. All groups received 0.5 ml of 0.5% bupivacaine.	It was 2 ml of normal saline. All groups received 0.5 ml of 0.5% bupivacaine.	NRS, ODI, adverse event	Transforaminal	–	–
ter Meulen et al.	2023	Intervention: 46 Control: 50	3, 6, 3, 6 months	1 ml of 40 mg/ml Methylprednisolone plus 1 ml of 0.5% Levobupivacaine	1 ml of 0.5% Levobupivacaine and 1 ml NaCl 0.9%	NRS, RMDQ, GPR, Surgery rate, Opioid use	Transforaminal	68%	59%

### Characteristics of population and interventions

This analysis included 11 articles with a total of 978 participants, including 485 in the test group and 493 in the control group. The test group used the following steroids: methylprednisolone (28–33), dexamethasone (34), betamethasone (35–38), the control group received normal saline and(or) local anesthetics. The injections of steroids were administered through four different routes: interlaminar, parasaggital interlaminar, transforaminal and caudal approach (Table 1).

### Pain relief

**Figure 2:**
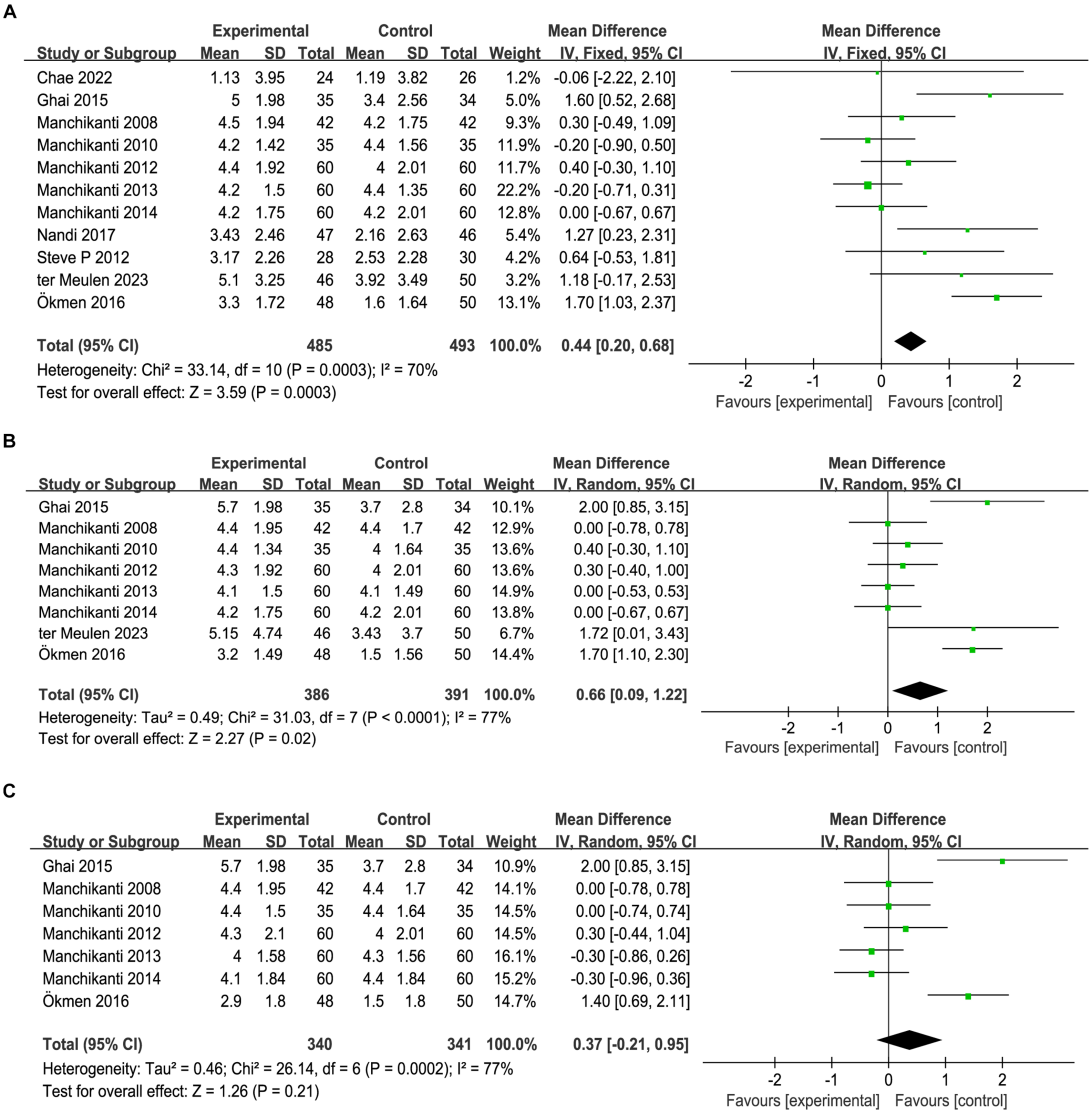
Meta-analysis of pain relief 3 months **(A)**, 6 months **(B)** and 12 months **(C)** after epidural steroid injection. Epidural steroid injection could significantly relieve sciatica in short- and medium-term.

Pain relief was reported in all 11 studies at the 3-month mark after intervention, but there was statistical heterogeneity observed among the studies (*I^2^* = 70%, *p* = 0.0003). A random effects model was used for meta-analysis, as shown in Figure 2A. The meta-analysis results indicated a statistically significant difference in pain relief between the two groups [MD = 0.44, 95%CI (0.20, 0.68), *p* = 0.0003], that is, in contrast to the control group, the administration of epidural steroid injection substantially mitigated pain. A total of 8 articles were identified in which the changes in NRS scores at 6 months after the intervention were documented. Due to the significant heterogeneity found in the data (*I^2^* = 77%, *p* < 0.0001), the random effects model was chosen to account for these differences and provide a more accurate estimate of the overall effect size, and the analysis results showed that epidural steroid injection could significantly relieve sciatica caused by lumbar disc herniation [MD = 0.66, 95%CI (0.09,1.22), *p* = 0.02] (Figure 2B).

Seven articles documented changes in the NRS 12 months after the intervention, heterogeneity test showed that there were heterogeneities between groups (*I^2^* = 77%, *p* = 0.0002). The results of the meta-analysis, using a random effects model, indicated that there was no statistically significant difference in the long-term reduction of sciatica between the epidural steroid injection group and the control group [MD = 0.37, 95%CI (−0.21, 0.95), *p* = 0.21] (Figure 2C), which demonstrates that the effect of epidural steroid injection on long-term relief of sciatica was minimal compared to the control group. Based on the above results, it is evident that epidural steroid injection plays a promising role in alleviating pain associated with sciatica over the short- and medium-term. However, its effectiveness diminishes when it comes to long-term pain relief.

### Function improvement

**Figure 3:**
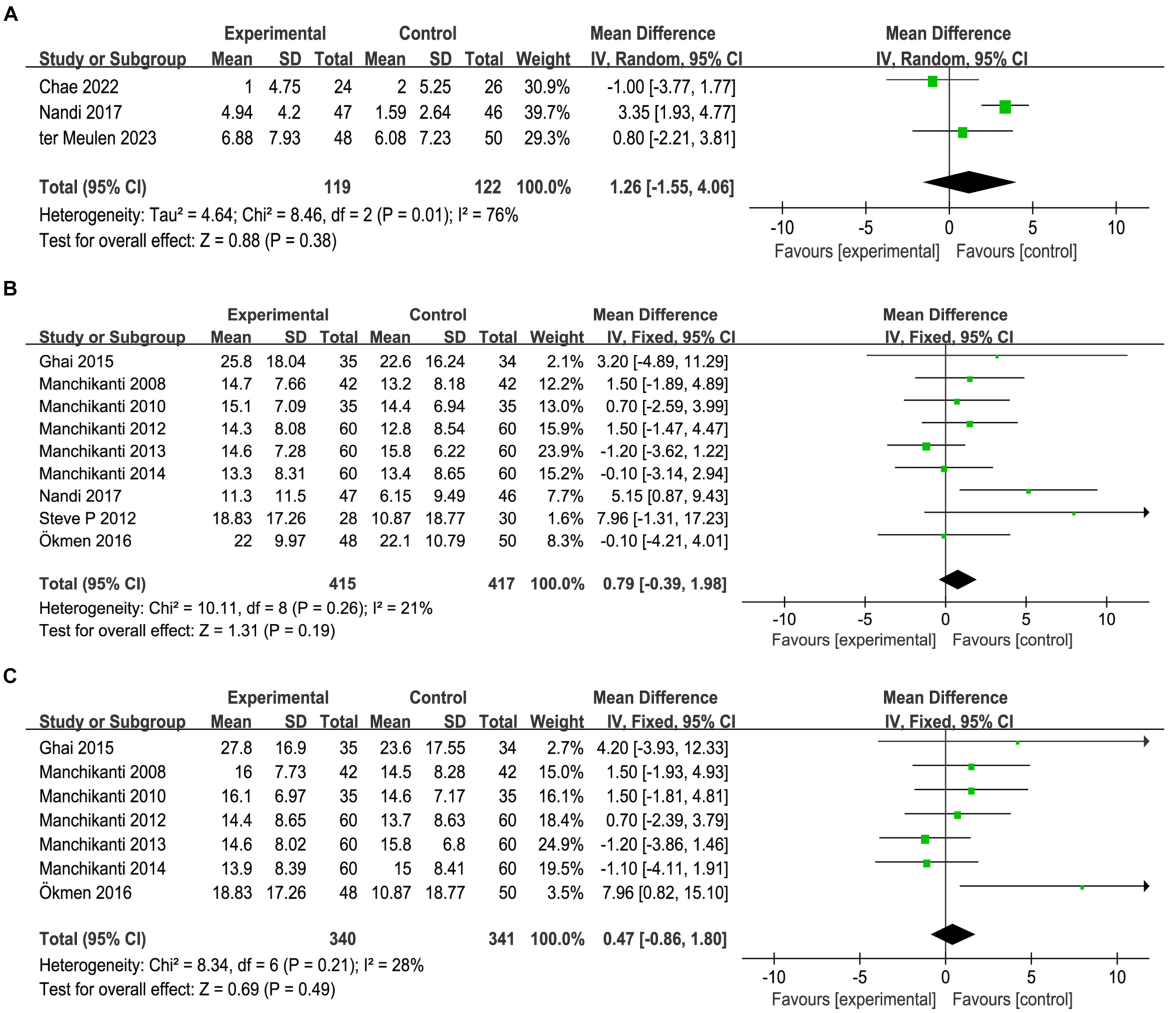
Meta-analysis of function recovery 1 months assessed by RMDQ **(A)** and ODI **(B)** and 12 months assessed by OID **(C)** after epidural steroid injections. Epidural steroid injection could not improve function of affected limb.

The changes in RMDQ before and 1 month after intervention were documented in three articles, and there were statistical heterogeneities among the studies (*I^2^* = 76%, *p* = 0.01), which might be attributed to the different dose used in Chae et al. (34) study, their dose was higher than the other two studies. A random effects model was used for meta-analysis, as shown in Figure 3A, the results showed that, compared with the control group, epidural steroid injection did not significantly improve the function of the affected limb in the short term [MD = 1.26, 95%CI = (−1.55, 4.06), *p* = 0.38]. Furthermore, 9 studies recorded changes in ODI before and 1 month after the intervention. A random effects model was employed for meta-analysis, which further corroborated the limited effectiveness of epidural steroid injection in promoting function recovery [MD = 0.79, 95%CI = (0.39, 1.98), *p* = 0.19] (Figure 3B). In total, 7 articles documented ODI changes prior to intervention and 12 months after intervention. Statistical heterogeneities were observed among the studies (*I^2^* = 28%, *p* = 0.21). A random effects model was employed for meta-analysis, as depicted in Figure 3C, and the findings were not statistically significant [MD = 0.47, 95% CI = (−0.86, 1.80), *p* = 0.49]. Therefore, it could be concluded that the use of epidural steroid injection does not demonstrate a substantial effect on functional improvement when compared to the control group.

### Opioid use

**Figure 4:**
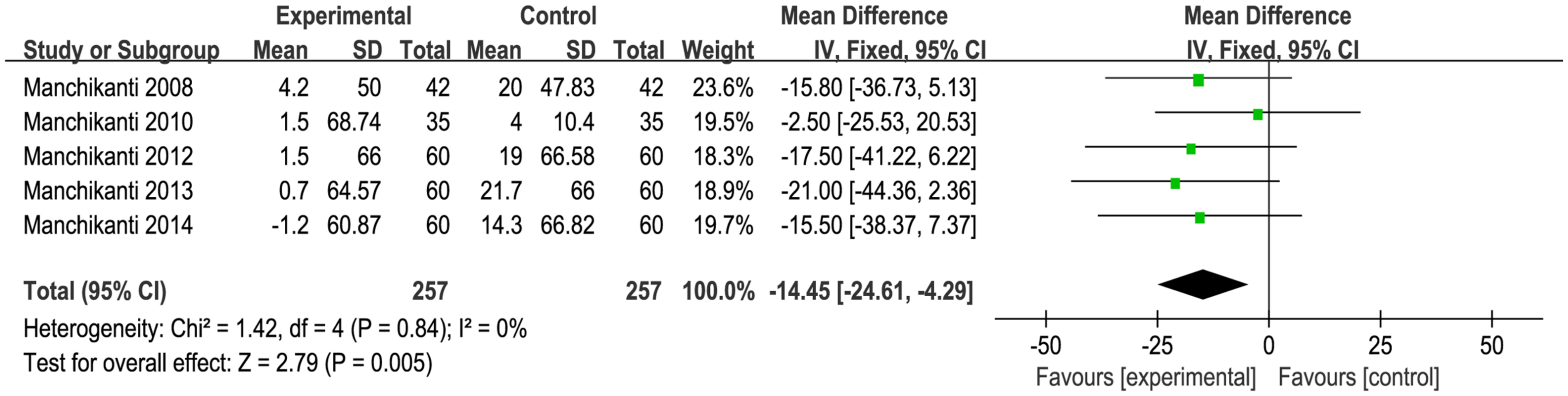
Meta-analysis of opioid use. Epidural steroid injection could significantly reduce opioid use for sciatica.

During the studies conducted by Manchikanti (35–38), the researchers documented the changes in opioid use both before and 3 months after the intervention. The results were summarized in Figure 4. As the studies were conducted by a single team using identical criteria, there was no statistical heterogeneity observed between the studies (*I^2^* = 0%, *p* = 0.84). Meta-analysis was conducted using the fixed-effect model, which revealed a significant reduction in opioid consumption among patients who received steroid injections [MD = −14.45, 95% CI = (−24.61, −4.29), *p* = 0.005].

### Adverse events

There were 8 articles reporting adverse events related to epidural injections. Possible adverse events included dural punctures, postdural puncture cephalalgia, infection, persistent paresthesias, new neurologic symptoms, systemic steroid reactions, skin lesions, or any adverse events to contrast media or adjuvant medications. Intravascular spread was also a potential adverse event (Table 2). The incidence of adverse events is low, varying from 0.3 to 3.5%.

**Table 2:** Adverse events reported.

**Study**	**Number**	**Description**
Chae 2022	0/54	–
Ghai 2015	2/69	Intravascular spread (2.9%)
Manchikanti 2008	0/84	–
Manchikanti 2010	1/283	Dural puncture, with no postoperative headache (0.3%)
Manchikanti 2012	0/120	–
Manchikanti 2013	5/714	4 subarachnoid punctures without headache (0.6%), 1 with nerve root irritation (0.1%),
Manchikanti 2014	28/601	28 intravascular infiltrations (4.6%), 9 nerve root irritations (1.5%).
Steve 2012	7/58	1 nonlocal rash (3.5%) in 28 patients in the experimental group, 3 nonlocal infection and 3 worsening pain in 30 control patients (20%).

### Quality assessment

**Figure 5:**
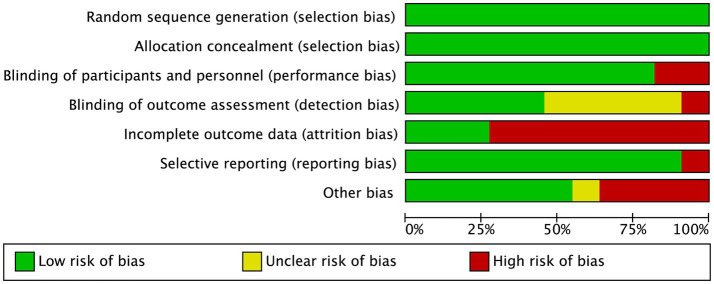
Risk of bias summary.

**Figure 6:**
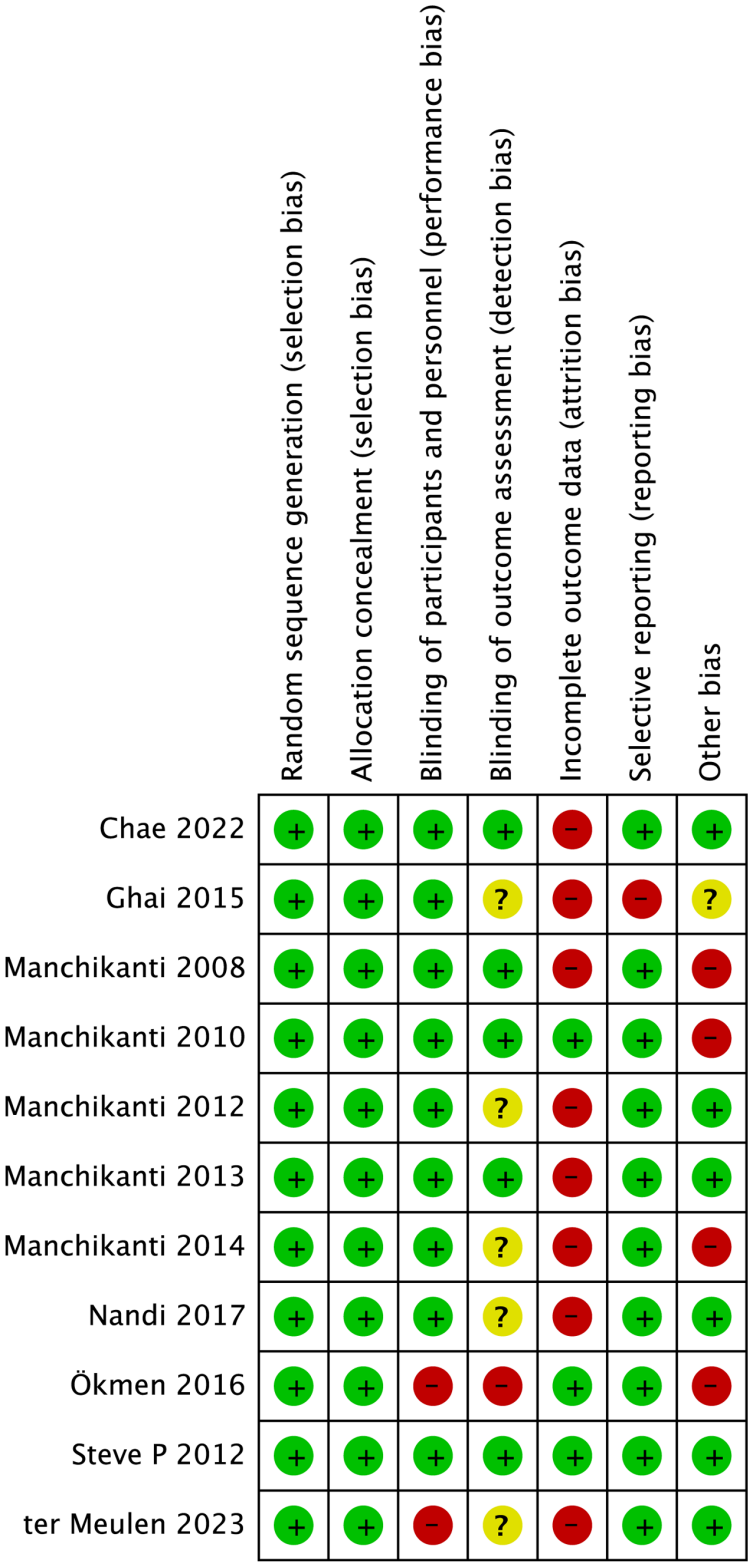
Risk of bias of studies included in the meta-analysis.

**Figure 7:**
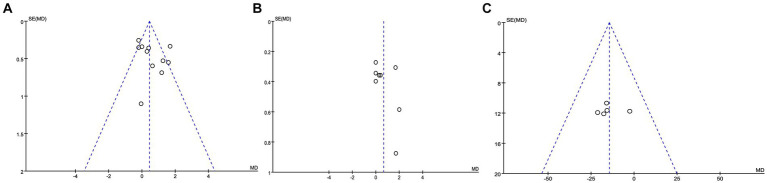
Funnel plots of reported outcomes. **(A,B)** Pain relief after 3 months **(A)** and 6 months **(B)** after epidural steroid injection; **(C)** opioid use.

Since all the studies analyzed were RCTs, we utilized Cochrane review criteria to assess their quality. Upon evaluation, it was found that only one article had all six indicators showing low risk, five articles had one indicator of high risk, four articles had two indicators of high risk, and one article had three indicators of high risk (Figure 5). Due to the common occurrence of loss of follow-up bias in RCT studies, incomplete outcome data was deemed high risk in all eight articles (Figure 6). Due to the limited number of trials included, the analysis of the funnel plot (Figure 7) for assessing publication bias presents certain constraints. Nevertheless, no decisive evidence suggesting the existence of publication bias was identified.

## Discussion

Lumbar disc herniation is the primary culprit in the development of sciatica, with significant evidence suggesting that besides nerve root compression, the intricate interplay between inflammation, immunity, and stress-related processes can contribute to the condition (39). Steroids have demonstrated a noteworthy ability to diminish inflammation, thwart the onset of immune and pathological immune responses, decrease swelling, and alleviate nerve root compression (40). As a result, steroid injections offer a viable treatment option for sciatica.

The findings of this study indicated that, compared to epidural injection of local anesthetics or normal saline, the use of steroids for epidural injection offered superior pain relief in the short- and medium-term, although this effect diminished over time. These results were predictable, as sciatica was known to be a self-limiting condition with a positive prognosis, and the temporary effect of steroids typically lasted for a few weeks to several months before gradually decreasing. Our study’s findings were consistent with prior researches in this field (25–27).The latest RCT study mentioned that the association between pain reduction after epidural injection and opioid use was worth further investigation, so we conducted a meta-analysis of opioid use (28). We found that the application of epidural steroid injections effectively decreased the usage of opioid among sciatica patients. This finding further confirmed the efficacy of epidural steroid injections in treating sciatica and demonstrated that the pain relief achieved was not solely derived from opioid use (41). While the long-term pain relief effect of epidural steroid injections might not have been substantial, the reduction in opioid usage indirectly impacted its pain relief effectiveness. As widely recognized, a reduction in opioid usage implied a lower incidence of drug-related complications (41, 42). Ultimately, this study’s results suggested that epidural steroid injections could effectively alleviate sciatica resulting from lumbar disc herniation.

As for functional improvement, the present work showed that there were no statistical functional improvements for epidural steroid injections in short- and long-term. The results supported the conclusion of the latest RCT study (28), but were sightly inconsistent with previous study which mentioned that epidural corticosteroid injections were slightly more effective for disability than placebo injections at short term follow-up (22). The possible reason for this could be that the RCTs included in this study enrolled patients who were in the early stages of lumbar disc herniation, experiencing only pain symptoms caused by nerve root irritation, without any significant impairment of lower limb function. During this phase, pain was the primary symptom and functionality was not affected. Therefore, after receiving epidural steroid injections, there was a significant reduction in pain symptoms but no noticeable improvement in limb function. Therefore, further analysis is required to assess the improvement in limb function after epidural steroid injection treatment in patients with impaired limb function, in order to objectively evaluate the impact of this treatment on pain relief and functional improvement.

In this study, the administration mode was described in detail. In terms of the proportion of patients with pain relief, the parasaggital interlaminar route had the best effect, followed by the caudal injection and interlaminar route, and the transforaminal approach had the worst effect, which was slightly inconsistent with the results of previous study (18). However, due to the small number of studies included in this study and the large differences in the proportion and component of drugs, this conclusion is not highly reliable.

When it comes to adverse events, the occurrence of adverse events is predominantly linked to the invasive procedure itself, rather than the substances being injected. It is vital to recognize that while the overall rate of complications from epidural injections is low, there is a wide range of adverse events, and some of them can be fatal. This emphasizes the need for healthcare practitioners to exercise caution and have essential emergency tools within reach by the patient’s bedside.

There are still certain limitations in this study. Firstly, the drug dosage and delivery methods were not standardized in this meta-analysis, instead, the study used time points that were as close as possible for combining, leading to heterogeneity in the results. Secondly, due to significant variations in intervention methods, the relationship between steroid dosage and its clinical efficacy was not further analyzed, to achieve better efficacy, future research should focus on exploring the optimal ratio of steroids and local anesthetics in injection contents. Thirdly, some of the included articles were assessed as high risk, resulting in low internal validity of the evidence. Finally, it might be clinically significant to analyze the effect of epidural corticosteroid injections in pain relief of acute, subacute and chronic sciatica, but all RCTs included in this study did not provide these details.

## Conclusion

In this meta-analysis, we showed that epidural steroid injection has demonstrated notable efficacy in relieving sciatica caused by lumbar disc herniation in short to medium-term, and reduce opioid use for sciatica, while there were not significant improvements on function of affected limb.

## Author contributions

JZ: Writing – original draft, Investigation. RZ: Writing – review & editing, Data curation. YW: Writing – review & editing, Software. XD: Writing – review & editing, Funding acquisition, Conceptualization.
