# Supplementary material for: Efficacy of epidural steroid injection in the treatment of sciatica secondary to lumbar disc herniation: a systematic review and meta-analysis
Source: Front Neurol. 2024 May 22;15:1406504. doi: 10.3389/fneur.2024.1406504 (PMC11150834; doi:10.3389/fneur.2024.1406504)
Supplement: Supplementary file 1 [file Table_2.DOCX]

(("Sciatica"[mesh] OR "Sciatica"[tw] OR "Lumbosacral radicular syndrome"[tw] OR "LRS"[tw] OR "Lumbar radicular syndrome"[tw] OR "Lumbosacral radicular pain"[tw] OR "LRP"[tw] OR "Sciatic neuralgia"[tw] OR "Sciatic neuralgias"[tw] OR "Bilateral sciatica"[tw] OR "Bilateral sciaticas"[tw] OR "Sciatic neuropathy"[mesh] OR "Sciatic neuropathy"[tw] OR "Sciatic neuropathies"[tw] OR "Low back pain"[mesh] OR "Lower back pain"[tw] OR "Low back pain" [tw] OR "Low back pains" [tw] OR "Lower back pains" [tw] OR "Low back ache" [tw] OR "Low back aches" [tw] OR "Low backache" [tw] OR "Low backaches" [tw] OR (("Spinal nerve pain"[tw] OR "Spinal nerve pains"[tw] OR "Pinched nerve"[tw] OR "Peripheral nerve compression"[tw] OR "Peripheral nerve compressions"[tw] OR "Spinal nerve compression"[tw] OR "Spinal nerve compressions"[tw] OR "Pinched nerve"[tw] OR "Pinched nerves"[tw] OR "Radiculopathy"[mesh] OR "Radiculopathy"[tw] OR "lumbar disc herniation" [tw] OR "radicular pain" [tw]) AND ("Lumbosacral Region"[Mesh] OR "Lumbar Vertebrae"[Mesh] OR "Lumbar"[tw] OR "Lumbosacral"[tw] OR "Lower back"[tw]))) AND ("Injections, Epidural"[mesh] OR "Epidural injection"[tw] OR "Epidural injections"[tw] OR "Extradural Injections"[tw] OR "Peridural Injections"[tw] OR "Peridural Injection"[tw] OR "Extradural Injection"[tw] OR "Vertebral injection"[tw] OR "Vertebral injections" [tw] OR "ESI"[tw] OR (injection*[tw] AND (Epidural*[tw] OR extradural*[tw] OR peridural*[tw])) OR "Intrathecal injection"[tw] OR "Intrathecal injections"[tw] OR (("Steroid injection"[tw] OR "Steroid injections"[tw] OR "corticosteroid"[tw] OR "corticosteroids"[tw] OR "Corticosteroid injection"[tw]) AND ("epidural"[tw] OR epidural*[tw] OR extradural*[tw] OR peridural*[tw]))) AND ("Placebos"[mesh] OR "Placebo"[tw] OR placebo*[tw] OR "sham injection"[tw] OR "sham injections"[tw] OR "saline"[tw] OR "sham"[tw] OR "anesthetic"[tw] OR "anaesthetic"[tw] OR "analgesic"[tw] OR "inactive"[tw]) AND ("Visual analog scale"[mesh] OR "Visual analog scale"[tw] OR "Visual analogue scale"[tw] OR "VAS"[tw] OR "Verbal rating scale"[tw] OR "VRS"[tw] OR "Verbal descriptor scale"[tw] OR "VDS"[tw] OR "Numeric rating scale"[tw] OR "NRS"[tw] OR "Pain reduction"[tw] OR "Pain relief"[tw] OR "Pain measurement"[mesh] OR "Pain measurement"[tw] OR "Oswestry disability index"[tw] OR "ODI"[tw] OR "McNab"[tw] OR "MacNab"[tw] OR "ODQ"[tw] OR "Oswestry Disability Index"[tw] OR "self-evaluation"[tw] OR "improvement"[tw] OR "recovery"[tw] OR "success"[tw] OR "leg pain"[tw]) AND ("clinical trial"[pt] OR "clinical trial"[tw] OR "clinical trials as topic"[mesh] OR "clinical trials"[tw] OR "control groups"[mesh] OR "control group"[tw] OR "control groups"[tw] OR "controlled clinical trial"[pt] OR "controlled clinical trials as topic"[mesh] OR "cross-over studies"[mesh] OR "cross over study"[tw] OR "cross over studies"[tw] OR "double-blind method"[mesh] OR "double blind"[tw] OR "evaluation studies as topic"[mesh] OR "follow-up studies"[mesh] OR "follow up study"[tw] OR "follow up studies"[tw] OR "placebos"[mesh] OR placebo*[tw] OR placebos*[tw] OR "pragmatic clinical trial"[pt] OR "prospective studies"[mesh] OR "prospective study"[tw] OR "prospective studies"[tw] OR "RaCT"[tw] OR "RaCTs"[tw] OR "random allocation"[mesh] OR "randomised "[tw] OR "randomized controlled trial"[pt] OR "randomized controlled trials as topic"[mesh] OR "randomized"[tw] OR random*[tw] OR "RCT"[tw] OR "RCTs"[tw] OR "Research Design"[MeSH:noexp] OR "Research design"[tw] OR "Research designs"[tw] OR "single blind"[tw] OR "single-blind method"[mesh] OR ((single*[tw] OR double*[tw] OR triple*[tw]) AND (blind*[tw] OR mask*[tw])) OR volunteer*[tw] OR "trial"[ti] OR "trials"[ti]))
